# Before the Lords' Committee

**Published:** 1890-07-19

**Authors:** 


### Before the Lords' Committee.

(By Our Special Commissioner.)

### VII.—NURSING AT THE LONDON HOSPITAL.

No one who has any real knowledge of hospital politics and arrangements has any doubt whatever as to who is at the bottom of the charges which have been brought against the nursing department of the London Hospital before the Lords' Committee. Anyone who had any doubts had only to go down to the Committee-room to enable them to clearly understand how the charges had been brought together and what the object of the real conductors of these proceedings in reality is.

### The Charges.

The charges have been brought by Miss Ellen Mary Yatman, formerly a paying probationer; Miss Mary Raymond, a paying probationer, and afterwards trained at the London; Miss Violet Dickinson, another paying probationer; Miss Dorothy Janet Page, a probationer for twelve months; the Rev. R. H. T. Valentine, formerly the chaplain at the London Hospital; and Miss Eliza M. Homersham, paying probationer for three months.

The first accusation was that sometimes probationers were put in charge of a ward in the London when they had been in the hospital for only three, four, six, or eight months, entirely at the matron's discretion. Inexperienced nurses very often have responsible duties in the wards, which may be injurious to the patients. These inexperienced probationers may have rendered clumsy attendance, and sometimes they may have done harm to the patients and retarded their recovery. Miss Yatman, who gave this evidence, was not definite as to how any harm was caused, nor did she give any instances. She went on to say that the nurses suffered in health from over-work, and they were generally over-tired. She was among the number to leave the hospital in consequence of this. Out of 160 or 170 nurses only 26 or 27 were certificated. The staff of nurses in the wards was insufficient, and the work had to be hurried over in consequence. Nurses were on duty from 7 a.m. to 9.20 p.m., with two hours off duty. As to the nurses' food, Miss Yatman stated that it was not good on the whole—the butter was good sometimes, and the milk good unless sour. The meat was not good as a rule, and it was very badly cooked. "There was not much the matter with the tea." Complaints, however, had no result. Night nurses had to provide themselves with their own crockery, and took their meals in the ward, where they made their tea and cooked their food at the ward fire. "They were kept pretty well on the run all night." The patients' food was on the whole good, but no improvement occurred in that of the nurses during the eighteen months that Miss Yatman was at the hospital. Sick nurses were not satisfactorily supplied with food, and she knew cases where diets ordered for sick nurses by the physician or surgeon had not been supplied. The night nurses had insufficient food, and the day nurses had too little for supper. The meals were not eatable, as all the food was badly cooked and badly served. Nurses often had to provide themselves with food and pay for it. The quality of the day nurses' food was bad, and the quantity of the night nurses' food was insufficient. The bread was stodgy, but as a rule good. Nurses had left because the work was so hard and the food was so bad. Adult helpless patients were washed at 4 a.m., and the children even earlier than this, sometimes at 2 a.m. Patients had breakfast at 6 a.m. Miss Yatman had charge of the women's medical ward after three months' residence in the hospital. "Nurses generally sat outside of the wards so that they could hear everything that happened in each ward." She knew of several instances where the insufficient supply of nurses in the wards led to bad results by which the patients suffered very much. The children were neglected, and in support of this statement Miss Yatman said that a child who had had its eye excised cried bitterly all night and suffered injury in consequence. When the nurse took it in her arms it stopped. Two operation cases for hare-lip also suffered from the same cause. There were too many paying probationers in the hospital, and they rendered very little assistance. The bulk of the nursing at the London Hospital fell on the probationers. There were twenty of them who had not been in the hospital a year, acting as staff nurses. Probationers were often sent out, too, to private cases. The wards at the London Hospital were overcrowded, the medical being more so as a rule than the surgical. Miss Yatman knew cases where patients got the wrong medicine due to hurrying over the work. The linen supply was defective; the patients were not supplied with towels, but had to bring them in with them. Those who had no towel used a common round towel, one being allowed to each ward. These towels were changed sometimes once and sometimes twice a-week. Miss Yatman had heard of cases of patients being dried with a sheet. The nurses performed housemaids' duties to a certain extent. Probationers were three months on day and three months on night duty, with one day off a month. Probationary nurses doing Sisters' work only received probationers' pay. Only some of the Sisters were certificated, and the uncertificated Sisters were often placed over certificated staff nurses. Sisters are paid the same as nurses. Miss Yatman understood that all the above evils exist at the present time. The advertisement of the private nursing home was misleading. Nurses were kept on duty with poisoned hands and arms contracted in nursing a pyæmia case. Miss Furnace was a case in point, and Nurse Scott was kept on duty for three weeks with a poisoned finger.

Miss Mary Raymond, Miss Dickinson, and Miss Page confirmed, agreed with, and corroborated Miss Yatman. Miss Raymond added that nurses had not sufficient protection when they were dismissed. They were often dismissed unjustly by the matron, as in the case of a nurse who was told to go away because she applied outside for medical advice. She described the case of a man who, after an anæsthetic, fell out of bed while he was under the influence of chloroform, because the wards were so under-nursed that nobody could be set apart to watch him. He was finally tied to the bed, so as to enable the nurses to go on with their other ward work. An extra nurse was asked for, but was not supplied. The nurses had to discharge menial duties, including cleaning out the bed-rooms and lavatories and slops. This witness attributed Miss Edwards' death to overwork and underfeeding. In the children's ward there were 56 cots and only three nurses.

The Chaplain (the Rev. R. H. Ballantine) gave evidence that Nurses Howes, Dorman, Black, Hume, and Page were unfairly treated. The nurses in the sick room had not sufficient attention. A nurse was sent away with his knowledge as convalescent with a temperature of 103 (Nurse Powell). Another nurse was sent away with scarlatina (Nurse Lawson), whilst Page was dismissed for consulting an outside doctor, having previously been kept on in an exhausted state. The doctor consulted was Dr. Anderson, one of the visiting physicians at the hospital at the time. More could have been done for nurse Sable who died last summer. The short term probationers were of little use—some almost worse than useless—constituting "utterly incompetent helps." The house governor had nothing to do with the nursing. The chaplain's whole case was that "nobody took care of the nurses, who felt aggrieved." "The matron is queen of the nurses."

Mr. Ballantine considered it "simple tyranny to say that a nurse may not spend two guineas on consulting a herbalist if she likes." The tea of the patients in the ward was made in one pot, the pot being taken round and a spoonful of every patient's tea put in it. The quarterly meetings of the governors at the London were "absolutely a farce." Miss Stocking, one of the nurses, was kept working a fortnight after she had seen a visiting physician—Dr. Fenwick. She was very ill, yet she was sent back on duty, and she got no redress until she left the hospital.

The Refutation of the Charges.

The Secretary (Mr. Roberts) gave evidence as to the food supply of the nurses. He put in reports by the house visitors, which proved that the food supply of the nurses, as well as the food supply of the hospital patients has always engaged the most anxious care of the House Committee, and that they are fully satisfied with the food supply in every way. The house visitors had dined with the sisters and nurses, and their reports extended from June 1st, 1886, to March 3rd, 1890. A letter was put in from Mrs. Oram, the mother of a probationer who was in the sick room, dated July 3rd, 1890. Mrs. Oram had read with indignation the evidence given by the discharged nurses to the effect that nurses were worked when they were ill, that they were not sufficiently attended, and did not have proper medical attention. Mrs. Oram says: "I should feel I was shirking a duty and certainly wanting in gratitude if I did not offer my testimony to show how utterly groundless the charge is. I cannot speak too strongly or too gratefully of the skill, care, or kindness which my daughter received as a nurse when ill and up to the time of her discharge, cured on the May 4th. Her diet was of the very best, both in quality and quantity; she had everything she possibly could have, even oysters and champagne. In short, she owes her life to the treatment she received at the London Hospital. From the beginning of her illness until I brought her home I spent four hours or from six to seven hours of each day in the hospital, where I went about a good deal, and never did I hear one grumbling word; sisters and nurses were all perfectly happy in their little world, and speaking in terms of affection of the matron. My son, a medical student attached to another hospital, who was there a good deal, will corroborate all I have written." The following letter, dated July 1st, signed by nearly all the Sisters, probationers, and nurses was handed in. "We all unite in condemning the conduct of those nurses who have so unjustly attacked the hospital arrangements, and express our warmest sympathy for you in the charges against the hospital. Our deepest thanks are due to you for all you have done to the nursing staff since you have been here." The secretary stated that during the last two and a-half years there had been no difficulty in getting a quorum of Governors, forty-five of whom attended, besides the members of the Committee.

Miss Lückes, the matron, said she had held the post for ten years, and had had four years' previous hospital experience. She was trained at Westminster, and had had three months' experience at the London, as night sister, besides experience in children's hospitals and various others. She had four assistant matrons under her at the London. On July 3rd, the total staff of nurses, including the private staff, was 243. Of these 23 were sisters fully appointed, 34 certificated nurses taking staff duty, 52 nurses in second year, and 23 paying probationers of the same class; 82 probationers were in their first year, 11 of whom had passed a satisfactory examination, and 23 had received previous training, 23 paying probationers, and 25 on private nursing staff. There were 1,600 applications last year from women who desired to become probationers. Miss Lückes denied that it was possible that a nurse had ever been sent away at a week's notice. There had been wonderfully little grumbling during the last ten years. The London is a very happy hospital. As regards the food in 1882, Miss Lückes sent in a very painful and strong report to the committee, because it was not good enough for the women who were doing the duty. Since 1886, when the Nursing Homes Kitchen was formed, the nurses at the London had been most satisfactorily fed, and particular attention is paid to the quality of the nurses' food and to any grounds for complaint. The dietary for the ward meals of the night nurses includes bread and butter, a herring, tea and sugar, eggs, sausages, and cold meat, with bacon, cakes, and a little jam tart. Things had improved during the last ten years beyond recognition. The amount of assistance in the wards and the number of the nursing staff was sufficient for the purpose. It was not true that owing to the insufficiency of nurses unqualified nurses were placed in charge of delicate and dangerous duties. Miss Lückes had never known of any case retarded by the insufficiency of the nursing. The number of nurses had increased since Nurse Yatman first entered as a probationer in 1883. The sisters being responsible for the nurses both to the doctor and matron would not suffer a probationer who was not competent. Thus the sister who reported Nurse Raymond stated she could not be responsible for the patients in the ward if such a nurse was left there, and so Raymond was immediately removed. The sister is responsible for the wards. Miss Lückes maintained that when the staff nurse was away the person left in charge was always duly qualified. The sisters really superintended the nursing. Miss Yatman was moved because the sisters complained that it was not safe to leave her in charge, and so she was moved to another ward and given less responsible duties. If a nurse was guilty of misconduct she was reported to the house governor and then suspended, the report coming before the next house committee. The alteration in Rule XI. giving the matron power to terminate the engagement of probationers was framed with the object of being beneficial to the probationer as well as easier to the committee. This Miss Lückes made out clearly in her evidence. Nurse Stocking broke the engagement herself. A letter was put in showing that she had been very happy whilst she was at the London, and had no complaint at all to make when she was down at the convalescent home at Dover. It was absolutely impossible that she could have been kept on night duty for a fortnight whilst suffering from gastric ulcer with constant vomiting. In 1886 Drs. Sutton and Fenwick, with Mr. Treeves, three members of the visiting staff, undertook to be responsible for the health of the nurses. They saw any nurse who complained of being unwell, and any sister who did not look well. In the case of Miss Page, it is not true that any objection was taken to her seeing Dr. Anderson, but to the manner in which it was done—she being under treatment by another member of the medical staff at the time. "Her engagement was terminated on the ground of her inefficiency, not because she had seen an outside doctor, as alleged. Miss Page was inefficient before she came to the London, and at the last appointment that she held in October, 1889, she went to a hospital at Chelsea, and was obliged to leave there before the following Christmas on the ground of inefficiency." Nurse Page had been employed at Dr. Barnardo's Home at Jersey. Dr. Barnardo states that she was most unsatisfactory, and had been dismissed from that home. The especial cases she had charge of as mentioned by Mr. Ballantine were cases where she had not the sole charge, the doctor and the sister taking the especial care of each of these cases. Nurse Page's dismissal was entirely owing to her inefficiency, and had nothing to do with her visit to Dr. Anderson. She had probably done her best, but lacked the necessary capacity for nursing. A number of paying probationers had made complaints to the Committee, to whom they always write when they have grievances of any sort. Nurse Page must have known that the authorities at the hospital were under the impression that she lacked capacity. A year would be enough to enable a probationer to become a thoroughly qualified nurse technically, but some women would take three or four years, some much less. With regard to Nurse Homersham the matron reported that she left the hospital, breaking her agreement, on account of her father's illness, but with a great deal of superfluous rudeness, without bringing any doctor's certificate or any evidence that her presence was required. Miss Lückes declared that under no circumstances would she delay, much less refuse, the immediate departure of any member of the nursing staff when summoned by urgent illness to her friends. [There is abundant testimony that this is the invariable custom at the London Hospital.] The Committee found that "Eliza Homersham proved a very unsatisfactory probationer, and ended by giving great trouble. Her services were not valuable, and, in addition to being essentially common and lacking in refinement, we had already received considerable evidence of her troublesome temper before the final outbreak, and nothing could have developed her into a good nurse. The manner of her leaving and the general trouble and annoyance caused by her excitable brother are the only things to regret in connection with the breaking of this engagement." With reference to the charge about the towels, Miss Lückes stated that she believed it to be absolutely impossible that any towel could be used from patient to patient, as stated by one of the witnesses. Each patient usually brought a towel, a piece of soap, and a comb, and each visiting day the friends exchanged the dirty towel for a clean one. The wards were stocked with towels for the purpose of supplying those patients who did not bring any of their own, and several patients on the first admission required many towels. It was against the rules for towels to be taken from one patient to another, and the arrangements for a sufficiency of towels for the patients are proper and adequate. Regarding the charges as to the children being disturbed at two a.m., and the patients at four a.m., Miss Lückes declared that after a careful inquiry she was convinced it was not the case. The rule in the hospital is that all patients should breakfast at six o'clock. The patients who awake up early had permission to get up and go to the lavatory if they wished to do so, but the gas was never to be turned on until six a.m. As to the children, any child who was awake would be washed, but those who were asleep would not be aroused for this purpose. In the case of the children washed early they would be under seven years of age, they would settle off to sleep at six o'clock in the evening, and, therefore, have had ten hours' sleep, supposing they were awake at four a.m. It was not a fact that, owing to an insufficiency of nurses, children were washed earlier than this. As to Nurse Lambert's scarlet fever case, she would give evidence herself on the charges made in her name. Miss Lückes considers uncertificated probationers are sometimes more versed in nursing than certificated nurses. As to Miss Yatman's specific complaint that sewer gas was coming from the basins, the house governor, on investigation, proved it to be an escape of ordinary coal gas. The average cost of maintaining a sister at the London is 11s. 3¾d. per week, a nurse or probationer costs 8s. 11¾d., and a servant 8s. 1½d. Paying probationers were not necessarily ladies; regular probationers are very often ladies. Miss Lückes believed that it was impossible that a nurse could have been sent away from the hospital when her temperature was 103, and gave good reasons for this belief. Nurse Lawson's scarlatina case was quite true; it was a circumstance that all regret, and it was fully reported to the Committee. The proportion of sisters to patients was 1 to 24 patients in 1880, and 1 to $26\frac{1}{10}$ in 1889. The proportion of nurses to patients was 1 to $5\frac{1}{7}$ patients in 1880, and 1 to every $3\frac{1}{4}$ patients in 1889. As to the charge of insufficient nursing in the children's ward, Miss Lückes pointed out that the cots were seldom full, and that it was not to be conceived that surgeons with the reputation of the leading men of the London hospital, in justice to their patients or themselves, would allow operations like that of hare lip to break down through insufficient nursing, without making grave complaints to the matron and the committee. Miss Lückes could not recall a single complaint since the new *régime* of nursing. On the 10th inst. letters were read from Nurses Scott and Furniss, entirely destroying the charges against the London Hospital authorities made in their names. It also was proved in evidence that Nurse Sable was a most satisfactory nurse, and that she died from diphtheria contracted from a patient. Miss Lückes declared that if economy was no object throughout the country she would like to see every nurse having more holidays, shorter hours, and better pay. These were the three legitimate means by which she hopes nurses will progress all over England.

